# Supplementary material for: A sub-wavelength Si LED integrated in a CMOS platform
Source: Nat Commun. 2023 Feb 16;14:882. doi: 10.1038/s41467-023-36639-1 (PMC9935894; doi:10.1038/s41467-023-36639-1)
Supplement: Supplementary file 1 — Supplementary Information [file 41467_2023_36639_MOESM1_ESM.pdf]

Supplementary information: A sub-wavelength Si LED integrated in a CMOS platform

Zheng Li[1*†], Jin Xue[1,2†], Marc de Cea[1], Jaehwan Kim[1], Hao Nong[3], Daniel Chong[3], Khee Yong Lim[3], Elgin Quek[3] and Rajeev J. Ram[1*]

[1]Research Laboratory of Electronics, Massachusetts Institute of Technology, Cambridge, MA 02139, USA.
[2]Present address: Institute of Microelectronics (IME), A*STAR, Singapore 138634.
[3]GlobalFoundries Singapore Pte. Ltd., Singapore 738406.

*Corresponding author(s). E-mail(s): zhli@mit.edu; rajeev@mit.edu;
[†]These authors contributed equally to this work.

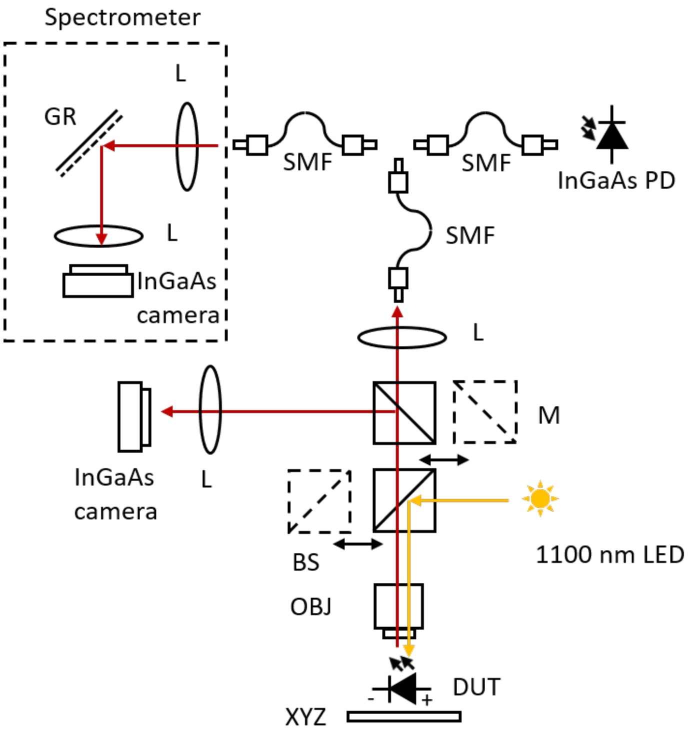

Supplementary Fig. 1: **Schematic of the microscope used in this study.** SMF: single-mode fiber; InGaAs PD: InGaAs photodiode; GR: grating; L: lens; M: mirror; BS: broadband beam splitter; OBJ: 0.95NA objective; XYZ: xyz piezo translation stage. The mirror and the beam splitter can be inserted and removed from the optical path.

1 Microscope

The LEDs were characterized using the microscope shown in Supplementary Fig. 1. The emission was collected using a 0.95NA objective and was routed either into an InGaAs camera for wide-field imaging or into a single-mode fiber (SMF) by flipping a mirror (M). Through the SMF, the emission was delivered into a photodiode or a spectrometer. A commercial 1100 nm LED was used as the illumination to take reflection micrographs.

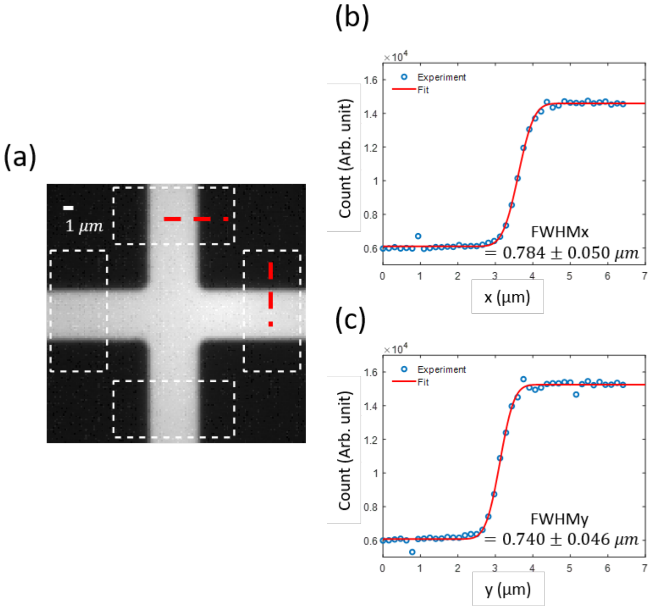

Supplementary Fig. 2: **Point-spread function (PSF) calibration.** (a) A micrograph of the reflective sample. (b, c) Two representative fit results on the red dashed lines in (a). The fit was repeated for all the edge profiles in the white dashed boxes in (a) and the standard deviations of the full-width-half-maximum (FWHM) were computed as the fit errors.

2 Point-spread-function calibration

The point-spread-function (PSF) of the microscope was calibrated using a reflective target sample with sharp edges. The edges were fit into erf functions which were used to extract the full-width-half-maximum (FWHM) of the PSF. The illumination was from a 1100 nm commercial LED to minimize the wavelength dependence of the PSF calibration.

3 Microscope transmission and camera calibration

The optical power transmission of the microscope was calibrated by back-propagating amplified spontaneous emission (ASE) of a semiconductor optical amplifier (SOA) from the collection SMF. An iris was used to match the beam size of the collimated ASE with the back aperture of the objective. The ASE spectrum is centered around 1130 nm, which is close to our emitter.

We measured the power of the ASE after the flipping mirror and after the objective. The optical power transmission is approximately 50.1% and 56.2% when the ASE is x-polarized and y-polarized, respectively. The polarization dependence is due to the two dichroic mirrors (not shown in Supplementary Fig. 1) between the objective and the flipping mirror. We used 53% as the average transmission to correct the emission power measured directly by the camera and the photodiode. Note that this correction does not compensate for the power loss from the fiber coupling and from the optics except the dichroic mirrors and the objective. The powers presented in the main text are therefore conservative estimations.

According to the factory test, the full well capacity and the ADC of our InGaAs camera are 1282000 and 16-bit, respectively, which corresponds to sensitivity of approximately 19.56 photo-electrons per digital count. The quantum efficiency is approximately 75% around 1100 nm. We thus can convert digital counts to photon numbers by 26.1 photons per digital count.

4 Gaussian fit and deconvolution

The images of the emission patterns were fit into two 2-D Gaussian functions with a constant.

$$I(x,y) = A_1 \exp\left(-\frac{(x-x_{0,1})^2}{2\sigma_{x,1}^2} - \frac{(y-y_{0,1})^2}{2\sigma_{y,1}^2}\right) + A_2 \exp\left(-\frac{(x-x_{0,2})^2}{2\sigma_{x,2}^2} - \frac{(y-y_{0,2})^2}{2\sigma_{y,2}^2}\right) + C \quad (1)$$

where the first Gaussian denotes the n+/n emission spot and the other two terms denote the background. The fit area is 20×20 μm^2. In the main text, we used the integral of the first Gaussian together with the optical power transmission and the camera sensitivity to estimate the n+/n emission power in Fig. 2 (e).

In Supplementary Fig. 3, FWHMs of the n+/n emission spot are presented. The curves labeled as fit are the Gaussian fit results from Supplementary Equation. 1, which are the convolution of the n+/n emission pattern and the PSF of the microscope. We assume both the emission pattern and the PSF are Gaussian-shaped and the deconvolved FWHM can thus be computed as

$$\mathrm{FWHM}_{x/y,\mathrm{decon}} = \sqrt{\mathrm{FHHM}^2_{x/y,\mathrm{fit}} - \mathrm{FHHM}^2_{x/y,\mathrm{PSF}}} \quad (2)$$

The deconvolved FWHMs are used to estimate the emission area in Fig. 2 (h).

From 1 mA to 6 mA the deconvolved FWHM decreases monotonically from 0.82 μm to 0.33 μm in x, while the FWHM in y remains approximately 0.5 μm below 4 mA and decreases to 0.35 μm at 6 mA. This trend is consistent with the carrier transport process in which the holes are confined by the electrical field from the Si filament and the localization becomes stronger with increasing bias. The deviation of the FWHM in y from the trend under low bias is probably because the holes are

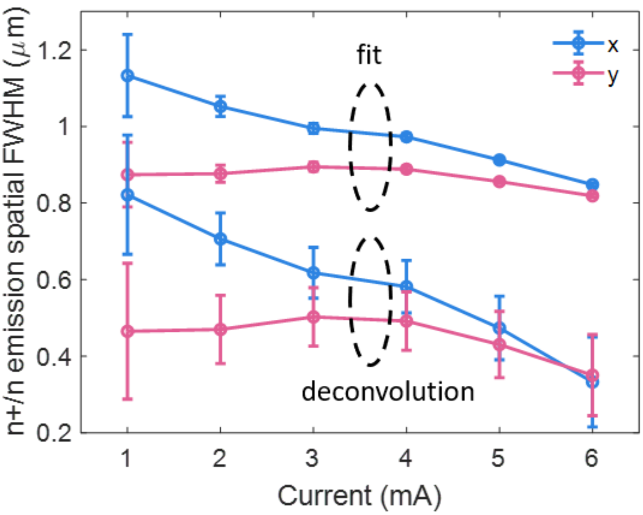

Supplementary Fig. 3: **Spatial full-width-half-maximum (FWHM) of the n+/n emission spot versus current.** The error bars are from the error propagation considering the PSF measurement error and the fit error.

confined by the STI in $\pm y$ instead of the field when the current is below 4 mA. While the STI does not exist in -*x*, the emission pattern is more extended in *x* when the bias is low. (Fig. 1 (c))

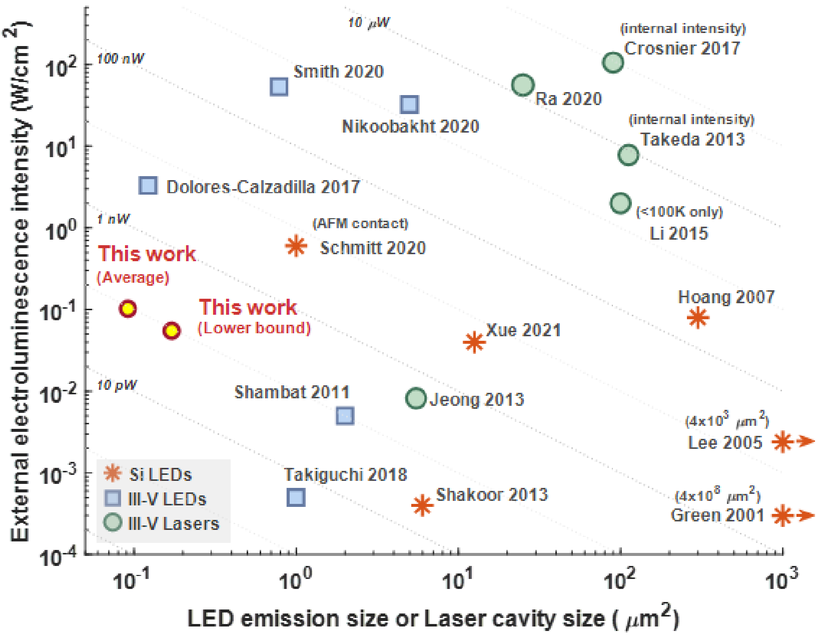

Supplementary Fig. 4: **Benchmark of the LED in this work compared with other related work.**

5 Benchmark of emission area and spatial intensity

The emission area of our LED is the smallest among the reported Si LEDs. The intensity is comparable with other state-of-the-art Si LEDs with much larger emission areas. The benchmark results are presented in Supplementary Fig. 4. The reference results are in [1–16].

6 Heat dissipation

We performed simulation of the heat dissipation in our device. In Supplementary Fig. 5 (a), we present the simulated structure of our device where we use homogeneous SiO_2, poly-Si, and crystalline Si to approximate the back-end-of-line dielectrics (BEOL), the n-poly-Si contact, and the substrate, respectively. The heat transfer equation is solved in 3-D assuming a cylindrical device geometry. We assume the bottom boundary of the substrate is fixed at 300 K and all the other boundaries are thermally isolated.

At the interface of the poly-Si and the c-Si, a 2-dimensional heat source with 300 nm diameter and 13.5 mW power is set. We estimate an upper bound for heat generation by assuming that the voltage across the device - excluding the n-poly-Si contact - is all on the Si filament and the heat exchange from the carriers to the lattice happens all in the active region. Specifically, at 6 mA, the measured total voltage is 5.9 V, the calculated voltage drop on the n-poly-Si contact is 3.6 V based on its geometry and sheet resistance, and hence the estimated voltage across the nanoscale emitter is 2.3 V. We also assume the active region is the same size as the n+/n emission spot (0.09 ± 0.04 μm^2 at 6 mA).

The simulated temperature distribution in the proximity of the heat source is presented in Supplementary Fig. 5 (b). The temperature spatially decreases to room temperature within 1 μm from the heat source and the maximum local temperature rise is approximately 170°C. Note that this is the upper limit of temperature rise in our device, which justifies that the substrate is an efficient heat sink.

As a comparison, we simulated an alternative device structure where SiO_2 is used to confine the current flow instead of a filament through the top oxide. In Supplementary Fig. 5 (c) we use the identical geometry of Supplementary Fig. 5 (a) but with a 0.3 μm thick SiO_2 layer around the heat source. This represents a design which has an active region of the same size of Supplementary Fig. 5 (a) but the carriers

are confined by shallow trench insulator (STI) instead of an electrical field. The simulated temperature distribution is presented in Supplementary Fig. 5 (d). Here the maximum local temperature rise is approximately 400°C, which is more than twice of the temperature rise in Supplementary Fig. 5 (b). We further increase the thickness of the STI to 0.5 μm and 1 μm for stronger carrier confinement, and the maximum local temperatures are approximately 550°C and 780°C, respectively. Strong Auger recombination and irreversible device degradation are likely to happen at these high temperatures.

The results above clearly show that efficient heat dissipation from the active region to a heat sink is required to ensure moderate local temperature rise. In our device, this is achieved by confining carriers using a local electrical field while leaving the heat conduction path to the substrate unaffected. The main limitation of the current design is that even though the lattice temperature rise is small the hot electrons injected from the n-poly-Si can still damage the device. This is likely the cause of the irreversible degradation at higher injection. We can further optimize the n-contact and fabricate shallow junctions near the active region to improve the reliability.

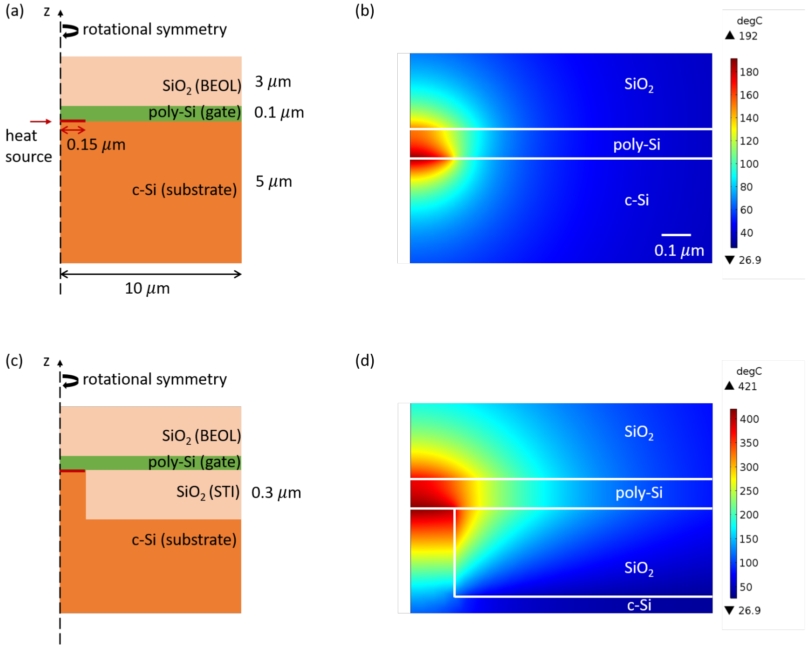

Supplementary Fig. 5: **Simulation results of heat dissipation.** (a) Simplified structure of our device assuming rotational symmetry around z axis. (b) Stationary temperature distribution of (a) near the heat source. (c) Device structure similar to (a) but with shallow trench insulator (STI) confinement. (d) Stationary temperature distribution of (c) near the heat source. The simulation was performed in COMSOL Multiphysics 5.3a. The thermal conductivities of c-Si, poly-Si, and SiO_2 are 156 W/(m·K) [17], 30 W/(m·K) [18], and 1.3 W/(m·K) [19], respectively.

7 Temperature-dependent device performance

We performed measurements on the SMF-coupled power with the chip die mounted on a temperature-controlled heat sink. (Supplementary Fig. 6.) We observe that the SMF-coupled power increases monotonically with temperature from 10°C to 70°C. A similar trend of emission enhancement has been reported by Ng et al. [20] where the emission increases with temperature from 80 K to approximately room temperature. This trend suggests that Auger recombination, which is usually the main cause of thermal droop, does not dominate the carrier recombination in the active region.

Multiple factors can contribute to emission enhancement with temperature. First, at the same current density, the electron concentration near the filament can increase with temperature because the electron mobility and the electron drift velocity in the filament decrease with temperature. Second, the hole concentration in the accumulation layer can also increase with temperature. The hole accumulation layer in our device is analogous to the inversion layer of a p-MOSFET of which the threshold voltage decreases with temperature [21]. If we assume the gate voltage (voltage between the n-poly-Si and the n-well in our device) and the gate capacitance do not change with temperature, the hole concentration increases. Moreover, as pointed out by Ng et al., the scattering rate of the confined carriers and the effective density of states increase with temperature, which can contribute to the increasing emission power.

Even though the device favors an elevated temperature near room temperature, we emphasize that heat dissipation is essential because the emission enhancement is based on the mitigation of Auger recombination which increases significantly with temperature. Moreover, we notice that at 85°C the optical power starts to decrease over time with 6 mA current, while the device is reliable with this current at 25°C. This suggests that breakdown site propagation, as a thermal runaway process, may happen with lower current at higher temperature.

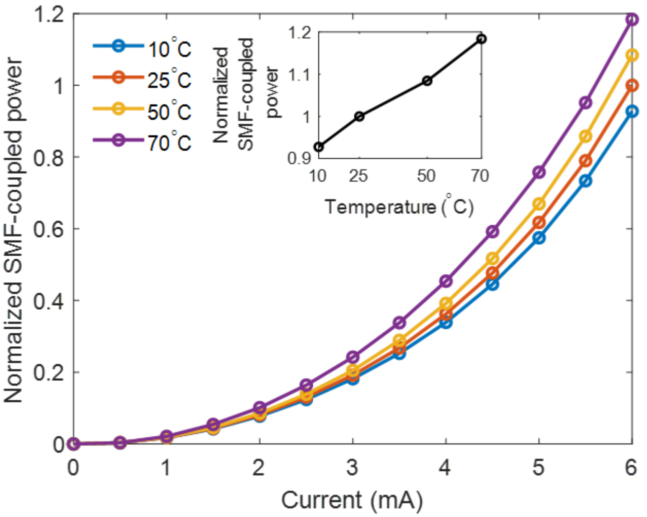

Supplementary Fig. 6: **Single-mode fiber (SMF)-coupled powers at multiple temperatures.** The data points are normalized by the power with 6 mA and at 25°C. The inset figure is the SMF-coupled power at 6 mA versus the heat sink temperature.

8 Variance and reliability

In Supplementary Fig. 7, the SMF-coupled powers and the forward bias voltages of five LEDs on different chip die are presented. These curves were measured after gate oxide breakdown and at room temperature. The relative standard deviations are presented in the inset figures. At 6 mA injection, the standard deviations of the SMF-coupled powers and the bias voltages are approximately 5.0 pW and 0.34 V, respectively, which are $\approx 13.7\%$ and $\approx 5.3\%$ of the mean values. These preliminary results indicate good reproducibility of our devices.

The good reproducibility of the silicon filament formation has also been reported in the literature. For example, these Si filaments (usually referred as anti-fuses in analog circuit communities) can be arrayed precisely in standard CMOS platforms as one-time programmable read-only memory (OTP-ROM). [22, 23]

The variance of our device is probably due to the spatial randomness of the breakdown site within the contact area of the n-well and the gate oxide ($\approx 0.3\ \mu\text{m}^2$). For example, if the breakdown happens near the interface of the n-well and the STI, a current leakage path may form. This is likely the situation of LED1 with the lowest SMF-coupled power since it also has the lowest forward bias voltage. We expect the reproducibility will be further improved with optimized design of the shape of the poly-Si contact.

In Supplementary Fig. 8, we plot the SMF-coupled power of LED2, which has intermediate performance in Supplementary Fig. 7 (a), after being turned on and off for $\approx 10^5$ times as a reliability test. After the test, the SMF-coupled power decreased by approximately 25%. No significant optical power decrease was observed in the following measurements. As we mentioned in the main text, this is probably due to lateral propagation of the breakdown sites, which also leads to bias voltage decrease.

In the main text, we mainly present the device performance of LED2 after the reliability test.

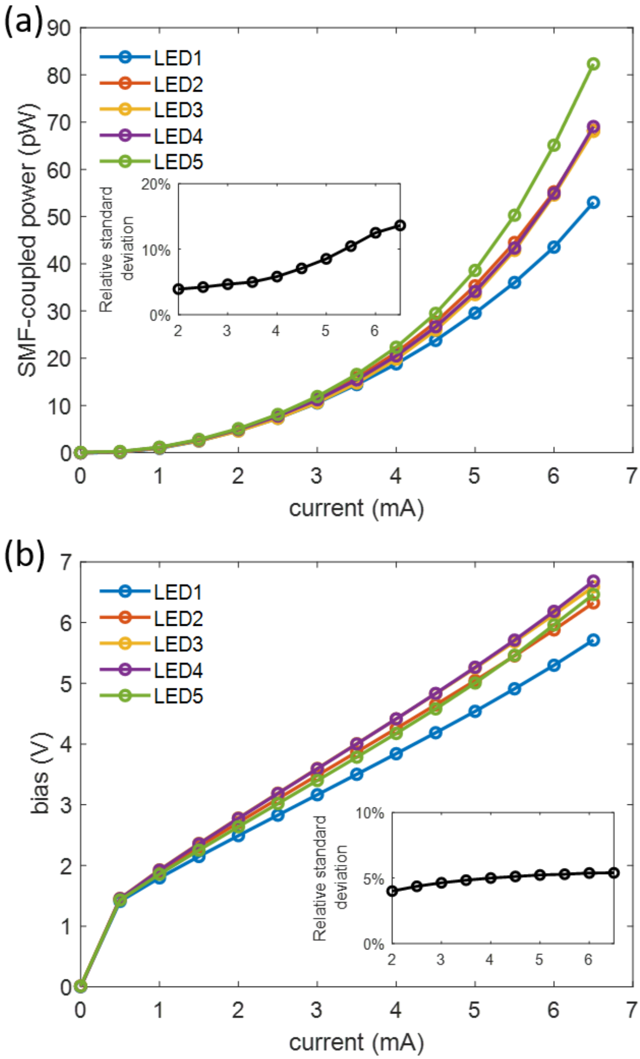

Supplementary Fig. 7: **Variance of device performance.** (a) Single-mode fiber (SMF) coupled power and (b) bias voltage versus current in five LEDs on different chip die after gate oxide breakdown.

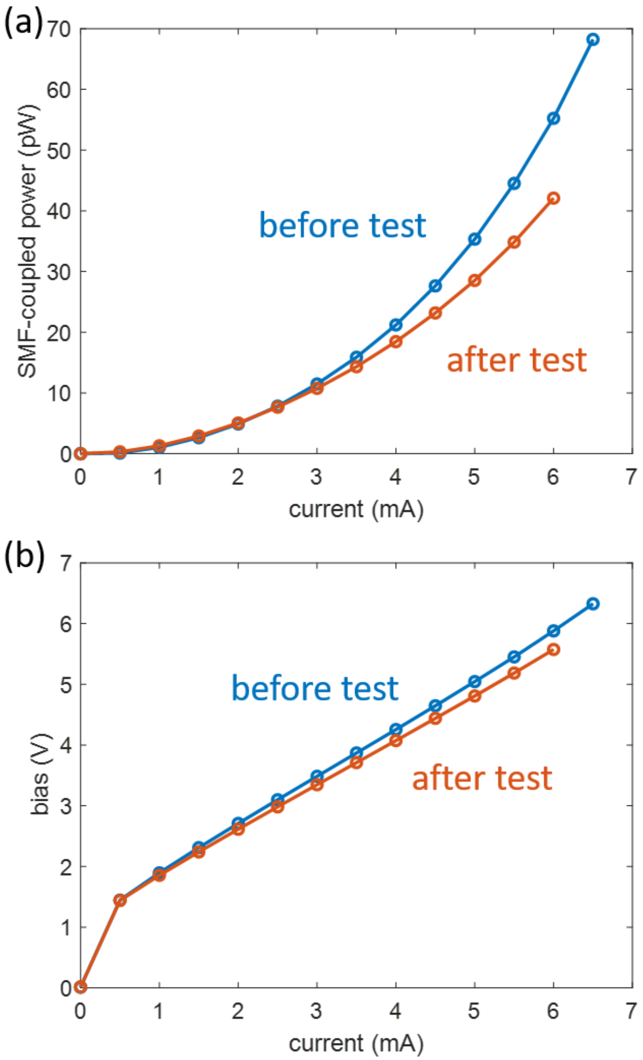

Supplementary Fig. 8: **Device reliability.** (a) SMF-coupled power and (b) bias voltage versus current of an LED before and after $\approx 10^5$ on/off switch.

9 Time-resolved optical power and modulation speed

We used time-correlated single-photon counting (TCSPC) technique to measure the time-resolved SMF-coupled optical power under rectangular voltage pulse. An arbitrary waveform generator (AWG, Agilent Technologies) was used to bias the LED and a silicon single photon avalanche diode (SPAD, Perkin Elmer), of which the quantum efficiency (QE) is $> 10\%$ at 1 μm, was used to detect optical pulses. The electronic signals were processed using a TCSPC timing module (B&H).

The TCSPC results are presented in Supplementary Fig. 9 (a, b). The optical pulses have distinct rise and fall edges. In Supplementary Fig. 9 (b), a 250 ns voltage pulse is applied and the LED reaches its steady state. The 10% - 90% rise and fall time are approximately 42.3 ns and 3.5 ns, respectively. The relatively long rise time is likely due to the diffusion capacitance of the substrate. In our current design, the contact to the p-substrate is approximately 2 mm away from the active region, which is not optimized for high-speed hole injection. Meanwhile, the fall time is relatively short because the holes can be swept out from the n-well by the built-in electrical field [24]. Similar carrier dynamics with asymmetric rise and fall edges have been reported in the literature. For example, in [25], Xu et al. report a Si electro-optic modulator based on a p-i-n ring resonator of which the carrier injection time (electrical rise time) is approximately 10 ns while the carrier extraction time (electrical fall time) is 100s of ps. In [26], Puliyankot et al. report fast-switching, forward-bias p-i-n LEDs fabricated in SOI process. The bandwidth (10 MHz) of their fastest device is limited by the relatively long rise time while the fall time can be neglected.

Although the rise time to the steady state is relatively long, the time from 10% to 50% is only 4.7 ns, which is comparable to the fall time. The rising edge of the optical power corresponds to a 3-dB switching bandwidth on the order of 100 MHz. In Supplementary Fig. 9 (c), we present the average optical power when the LED is modulated by square waves with 50% duty cycle and 50% DC offset. The

DC component (average) of the optical power was measured by a low bandwidth (< 20 Hz) photodiode (HP). Since the fall time is shorter than the rise time, the average optical power is mainly determined by the rising edge. With $0-4$ V and $0-3$ V voltage swing, the 3-dB bandwidths are 77 MHz and 51 MHz, respectively. These bandwidths are one order of magnitude higher than those reported in [26]. The fast modulation is a result of the small active region and we expect higher bandwidths from future designs since our current device is not optimized for high speed modulation.

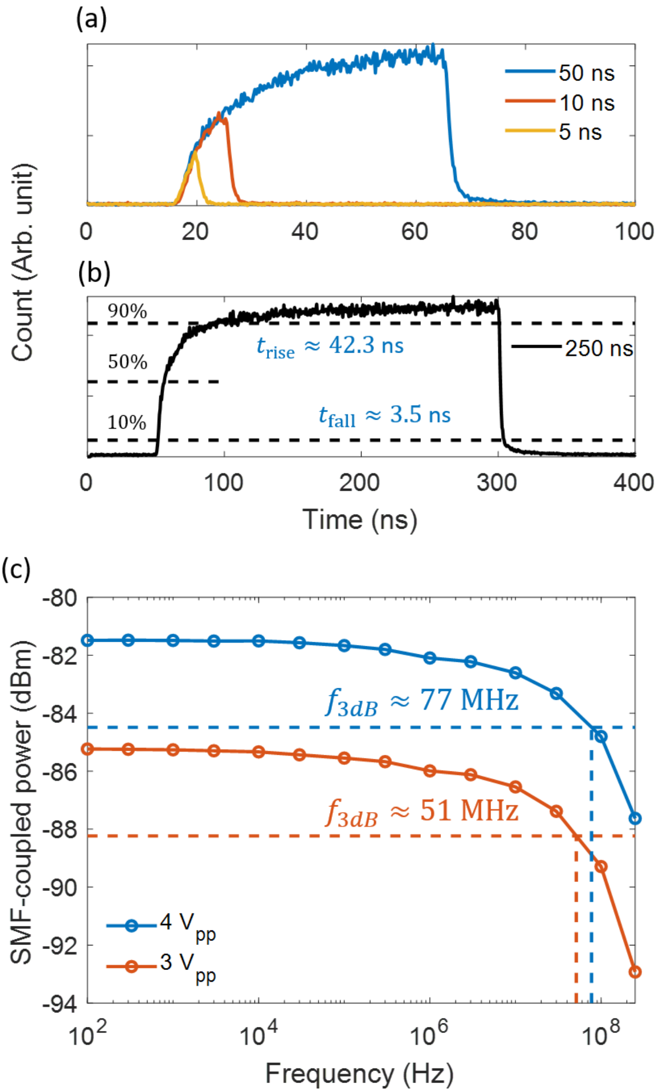

Supplementary Fig. 9: **Time-resolved optical power and modulation speed.** (a, b) Time-resolved SMF-coupled optical power under rectangular voltage pulse. The pulse is 0 – 4 V and the pulse widths are (a) 5 ns, 10 ns, 50 ns, and (b) 250 ns. In (b), 10%, 50%, and 90% of the steady-state power level are indicated by the dashed black lines. The 10% – 90% rise and fall time are also labeled. (c) Average SMF-coupled optical power with the bias modulated by 50% duty cycle square waves. The voltage swings are 0 – 4 V and 0 – 3 V.

10 Reference emitter

In Supplementary Fig. 10, the characterization results of the reference emitter are presented. Supplementary Fig. 10 (a) is a micrograph of the reference emitter biased at 0.9 mA taken by our microscope. Supplementary Fig. 10 (b) shows the emission pattern. The emission can be fit into a 2D Gaussian function of which the FWHMs are approximately 1 μm.

Supplementary Fig. 10 (c) shows the emission spectra at various currents. As discussed in the main text, compared with the LED, the reference emitter has a much broader emission spectrum, which indicates that the emission is mainly from impact ionization and hot carrier transition.

Supplementary Fig. 10 (d) shows the SMF-coupled power and bias voltage versus current of the reference emitter. The IV curve of the reference emitter is approximately linear and the resistance is approximately 20 kΩ. The SMF-coupled power has a current threshold, which probably corresponds to the bias voltage at which the hot holes gain high enough kinetic energy to introduce impact ionization. Compared with the characterization results of the LED, the reference emitter has different dominant transport and emission mechanisms.

Although most of the reference emitters could be biased up to approximately 1 mA, we noticed that the emission power started to degrade when the current was around 0.9 mA. Similar to the LEDs, this is likely due to lateral propagation of breakdown sites.

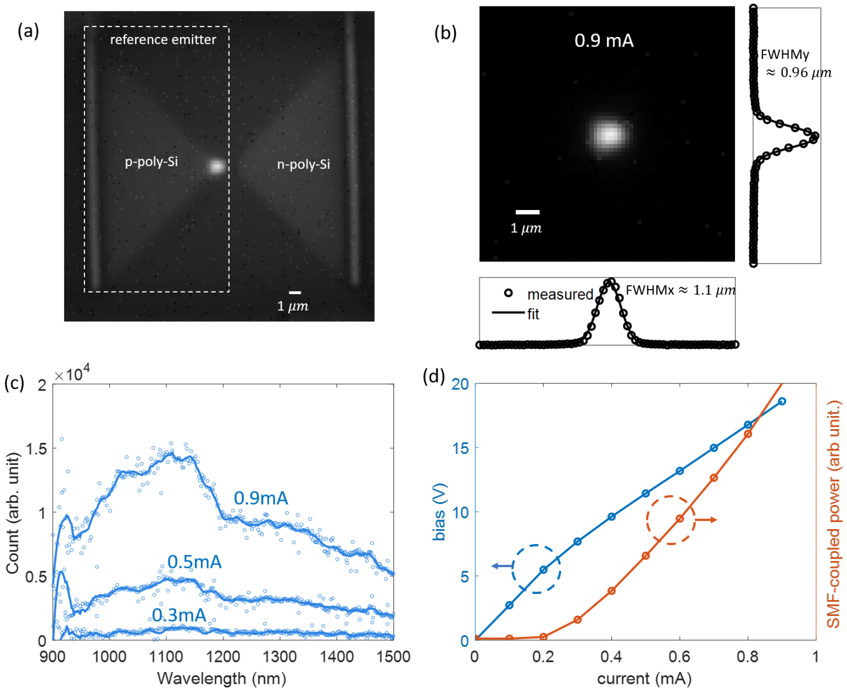

Supplementary Fig. 10: **Characterization of the reference emitter.** (a) Micrograph; (b) emission pattern; (c) emission spectra; (d) SMF-coupled power and bias versus current.

Supplementary References

[1] Green, M. A., Zhao, J., Wang, A., Reece, P. J. & Gal, M. Efficient silicon light-emitting diodes. *Nature* **412** (6849), 805–808 (2001) .

[2] Lee, H.-C. & Liu, C.-K. Si-based current-density-enhanced light emission and low-operating-voltage light-emitting/receiving designs. *Solid-state electronics* **49** (7), 1172–1178 (2005) .

[3] Shakoor, A. *et al.* Room temperature all-silicon photonic crystal nanocavity light emitting diode at sub-bandgap wavelengths. *Laser & Photonics Reviews* **7** (1), 114–121 (2013) .

[4] Xue, J. *et al.* Low-voltage, high-brightness silicon micro-LEDs for CMOS photonics. *IEEE Transactions on Electron Devices* **68** (8), 3870–3875 (2021) .

[5] Shambat, G. *et al.* Ultrafast direct modulation of a single-mode photonic crystal nanocavity light-emitting diode. *Nature communications* **2** (1), 1–6 (2011) .

[6] Dolores-Calzadilla, V. *et al.* Waveguide-coupled nanopillar metal-cavity light-emitting diodes on silicon. *Nature Communications* **8** (1), 1–8 (2017) .

[7] Takiguchi, M. *et al.* Direct modulation of a single InP/InAs nanowire light-emitting diode. *Applied Physics Letters* **112** (25), 251106 (2018) .

[8] Nikoobakht, B. *et al.* High-brightness lasing at submicrometer enabled by droop-free fin light-emitting diodes (LEDs). *Science advances* **6** (33), eaba4346 (2020) .

[9] Smith, J. M. *et al.* Comparison of size-dependent characteristics of blue and green InGaN microleds down to 1 μ m in diameter. *Applied Physics Letters* **116** (7), 071102 (2020) .

[10] Jeong, K.-Y. *et al.* Electrically driven nanobeam laser. *Nature communications* **4** (1), 1–6 (2013) .

[11] Takeda, K. *et al.* Few-fJ/bit data transmissions using directly modulated lambda-scale embedded active region photonic-crystal lasers. *Nature Photonics* **7** (7), 569–575 (2013) .

[12] Li, K., Liu, X., Wang, Q., Zhao, S. & Mi, Z. Ultralow-threshold electrically injected AlGaN nanowire ultraviolet lasers on Si operating at low temperature. *Nature nanotechnology* **10** (2), 140–144 (2015) .

[13] Crosnier, G. *et al.* Hybrid indium phosphide-on-silicon nanolaser diode. *Nature Photonics* **11** (5), 297–300 (2017) .

[14] Ra, Y.-H. *et al.* An electrically pumped surface-emitting semiconductor green laser. *Science advances* **6** (1), eaav7523 (2020) .

[15] Schmitt, S. W. *et al.* All-silicon polarized light source based on electrically excited whispering gallery modes in inversely tapered photonic resonators. *APL Materials* **8** (6), 061110 (2020) .

[16] Hoang, T., LeMinh, P., Holleman, J. & Schmitz, J. Strong efficiency improvement of SOI-LEDs through carrier confinement. *IEEE Electron Device Letters* **28** (5), 383–385 (2007) .

[17] Glassbrenner, C. J. & Slack, G. A. Thermal conductivity of silicon and germanium from 3 K to the melting point. *Physical review* **134** (4A), A1058 (1964) .

[18] McConnell, A. D., Uma, S. & Goodson, K. E. Thermal conductivity of doped polysilicon layers. *Journal of Microelectromechanical Systems* **10** (3), 360–369

(2001) .

[19] El-Kareh, B. & Hutter, L. N. *Fundamentals of semiconductor processing technology* (Springer Science & Business Media, 2012).

[20] Ng, W. L. *et al.* An efficient room-temperature silicon-based light-emitting diode. *Nature* **410** (6825), 192–194 (2001) .

[21] Wang, R., Dunkley, J., DeMassa, T. A. & Jelsma, L. F. Threshold voltage variations with temperature in MOS transistors. *IEEE transactions on Electron Devices* **18** (6), 386–388 (1971) .

[22] Kim, J. & Lee, K. Three-transistor one-time programmable (OTP) ROM cell array using standard CMOS gate oxide antifuse. *IEEE Electron Device Letters* **24** (9), 589–591 (2003) .

[23] Ng, K., Lee, M., Kwong, K. & Chan, M. Diode based gate oxide anti-fuse one time programmable memory array in standard CMOS process. *2009 IEEE International Conference of Electron Devices and Solid-State Circuits (EDSSC)* 457–460 (2009) .

[24] Schubert, E. F. *Light-emitting diodes* (E. Fred Schubert, 2018).

[25] Xu, Q., Schmidt, B., Pradhan, S. & Lipson, M. Micrometre-scale silicon electro-optic modulator. *Nature* **435** (7040), 325–327 (2005) .

[26] Puliyankot, V., Piccolo, G., Hueting, R. J. & Schmitz, J. Toward GHz switching in SOI light emitting diodes. *IEEE Transactions on Electron Devices* **65** (10), 4413–4420 (2018) .
